# Role of oxaliplatin combined with 5-fluorouracil and folinic acid in the first- and second-line treatment of advanced colorectal cancer

**DOI:** 10.3747/co.v13i5.99

**Published:** 2006-10

**Authors:** D. Jonker, R.B. Rumble, J. Maroun, the Gastrointestinal Cancer Disease Site Groupof Cancer Care Ontario’s Program in Evidence-Based Care

**Keywords:** Oxaliplatin, folfox, bevacizumab, 5-fu, fa, advanced colorectal cancer

## Abstract

**Question:**

What is the role of oxaliplatin combined with 5-fluorouracil (5-fu) and folinic acid (fa) in the first- and second-line treatment of advanced (unresectable locally advanced or metastatic) colorectal cancer?

**Perspectives:**

Evidence was selected and reviewed by two members of the Gastrointestinal Cancer Disease Site Group (gi dsg) of Cancer Care Ontario’s Program in Evidence-Based Care (pebc) and by a methodologist. The resulting practice guideline report has been reviewed and approved by the gi dsg, which comprises medical and radiation oncologists, surgeons, a pathologist, and patient representatives.

External review by Ontario practitioners was obtained through a mailed survey. Final approval of the original guideline report was obtained from the Practice Guidelines Coordinating Committee.

**Outcomes:**

Outcomes of interest were 1-year survival, response rates, and quality of life.

**Methodology:**

The medline, cancerlit, embase, Guidelines International Network, and Cochrane Library databases were systematically searched for relevant studies. Recommendations were formed based on the evidence reviewed. Through a survey, these recommendations were appraised by Ontario clinicians; the recommendations were then revised by the gi dsg. The systematic review and modified recommendations were approved by a review body within pebc.

**Results:**

The literature review found twenty-one randomized controlled trials and two meta-analyses. Evidence on first-line treatment found infusional 5-fu/fa/oxaliplatin (folfox) to be superior to bolus 5-fu/fa/irinotecan (ifl) for rates of median survival and tumour response, with lower incidences of most adverse effects except peripheral neuropathy. For second-line treatment after fluoropyrimidine monotherapy, folfox is a reasonable alternative for patients with contraindications to second-line irinotecan. After progression on infusional 5-fu/fa/irinotecan (folfiri), folfox is the preferred therapy. Evidence from a single randomized trial suggests that additional benefits can be expected with the addition of bevacizumab to the folfox regimen in second-line treatment.

**Practice Guideline:**

These recommendations apply to adult patients with advanced colorectal cancer who have high performance status (Eastern Cooperative Oncology Group score 0–2).

Refer to Appendix A for available treatment options and to Appendix b for recommended dosages and schedules.

The folfox regimen is an important component of therapy for advanced colorectal cancer.

**First-line Therapy**

In one trial, folfox was shown to be superior to ifl. The folfox regimen has superior rates of median survival and tumour response. Compared with ifl, folfox has lower incidences of severe nausea, vomiting, diarrhea, and febrile neutropenia, but a higher incidence of peripheral neuropathy.

Short-term infusional 5-fu/fa in combination with either oxaliplatin (folfox) or irinotecan (folfiri) are both acceptable alternatives for fit patients when combination therapy is the preferred treatment. Choice of first-line therapy may rely on patient factors and preferences—for example, less neuropathy with irinotecan versus less alopecia with oxaliplatin.

**Second-line Therapy**

After progression on first-line anti-thymidylate synthase monotherapy (for example, 5-fu/fa, capecitabine), irinotecan is standard second-line therapy. The folfox regimen is a reasonable alternative for patients with contraindications to the use of second-line irinotecan.

After progression on both irinotecan and an anti-thymidylate synthase agent, folfox is the preferred therapy. Recent trials suggest that, as compared with folfox alone, folfox combined with bevacizumab provides additional survival benefits.

**Qualifying Statements:**

The role of radiation therapy, either alone or in combination with chemotherapy, for locally advanced unresectable colorectal cancer is not addressed in this guideline.

Use of chronomodulated regimens is a topic that intersects with the use of oxaliplatin/5-fu combinations, particularly chronomodulation of 5-fu in these combinations. Chronomodulation of oxaliplatin has not been extensively studied, and the topic of chronomodulation is beyond the scope of this guideline and is not addressed.

Although data exist to support the use of bevacizumab in combination with folfox in second-line treatment, no first-line treatment data are available on which to make a recommendation.

## 1. QUESTION

What is the role of oxaliplatin combined with 5-fluorouracil (5-fu) and folinic acid (fa) in the first- and second-line treatment of advanced (unresectable locally advanced or metastatic) colorectal cancer?

Outcomes of interest were 1-year survival, response rates, and quality of life (qol).

## 2. CHOICE OF TOPIC AND RATIONALE

In Ontario, Canada, colorectal cancer is the fourth most common cancer site for both sexes combined, representing 13.1% of all new cancer cases 1. Colorectal cancer is the third most common site in men (13.3% of all new cases) and the second most common site in women (12.9% of all new cases) 1, and it remains the second leading cause of cancer death (10.6% of all cancer deaths) 1. Considering both men and women, colorectal cancer ranks third as the leading cause of death after breast and lung cancers in females and after lung and prostate cancers in males 1. For that reason, interest in improving the treatment results for this group of patients is great.

Currently, the standard first-line treatment for metastatic colorectal cancer (mcc) in Canada is a combination of 5-fu, fa [also known as leucovorin calcium (lv)], and irinotecan—called folfiri—delivered by infusion (Douillard regimen)^2^. Infusional folfiri replaced ifl, which is the same drug combination by bolus delivery. For patients unable to tolerate a combination-therapy regimen, an alternative to folfiri is monotherapy with a first-line thymidylate synthase (ts) inhibitor such as 5-fu/lv, raltitrexed 3, or capecitabine, followed by second-line irinotecan alone 4.

Once patients are no longer responding to the combined use of a ts inhibitor and irinotecan or to monotherapy, the options for treatment are limited. Oxaliplatin, a third-generation platinum compound has demonstrated activity in colorectal cancer. Oxaliplatin differs from both cisplatin and carboplatin in its amino acid configuration. Oxaliplatin has an oxalato group that is removed by hydrolysis and replaced with a diaminocyclohexane (dach) group^5^. The bulky dach side groups inhibit dna base excision by mismatching the repair enzymes 5. Because repair enzymes are particularly active in colorectal cancer, oxaliplatin has the potential to be of great benefit to patients in both first- and second-line treatment. With the availability of randomized trials comparing combination regimens of oxaliplatin and 5-fu/fa with other combinations, a systematic review of the evidence and clinical practice guideline were warranted.

## 3. METHODS

### 3.1 Guideline Development

This systematic review was developed by the Gastrointestinal Cancer Disease Site Group (gi dsg) of Cancer Care Ontario’s Program in Evidence-Based Care (pebc), using the methods of the practice guidelines development cycle 6. Evidence was selected and reviewed by two members of the gi dsg and by a methodologist.

The body of evidence in this review is primarily composed of data from mature randomized controlled trials (rcts). The systematic review and clinical practice guideline are intended to promote evidence-based practice in Ontario, Canada. The pebc is editorially independent of Cancer Care Ontario and the Ontario Ministry of Health and Long-Term Care.

### 3.2 Literature Search Strategy

A systematic search of the medline [1966 to June (week 1) 2006], cancerlit (1975 to October 2002), embase (week 26, 2003, to week 23, 2006), Guidelines International Network, and Cochrane Library (through Issue 1, 2006) databases was conducted in June 2006. The Medical Subject Heading search terms “colonic neoplasms,” “rectal neoplasms,” and “colorectal neoplasms” were combined with the text words “oxaliplatin”, “l-ohp,” “lohp,” and “folfox”. The results from that search were then combined with the terms “random” and “clinical trial” describing specific study designs. Results were limited to the English language. The conference proceedings of the 1999 to 2006 annual meetings of the American Society of Clinical Oncology, including the 2004 through 2006 gastrointestinal cancer symposia, were also searched for reports of new or ongoing trials. The reference lists from retrieved papers were searched for additional trials.

### 3.3 Study Selection Criteria

Articles were selected for inclusion in this systematic review of the evidence if they were

- phase iii rcts of oxaliplatin (l-ohp) combined with 5-fu/fa as first-line or second-line therapy for advanced colorectal cancer.
- abstract reports of trials.
- English-language published reports.

### 3.4 Synthesizing the Evidence

For the following reasons, the gi dsg decided not to pool the results of the trials found in the literature search:

- Treatments described were too heterogeneous to allow for pooling.
- Evidence from the studies obtained provided a clear indication of benefit or harm.
- Published meta-analyses of individual patient data were available. (The meta-analyses are discussed in the appropriate sections of this report.)

## 4. RESULTS

### 4.1 Literature Search Results

The literature search found thirty-three reports 7–39, including twenty-seven reports on randomized controlled trials (rcts) of first-line treatment 7–16,18–27,31–37, involving seventeen separate rcts 7–16,31–37; two meta-analyses on first-line treatment 28,29; and four reports on second-line treatment 17,30,38,39. Ten of these reports were preliminary publications that provided additional information about the included trials 18–27. Of the thirty-three trial reports obtained, twelve were fully published 7,8,10,11,15,16,28,31,32,34,35,37, and twenty-one were available in abstract form only 9,12–14,17–27,29,30,33,36,38,39. Of the seventeen main reports on first-line treatment, six were available as abstracts only 9,12–14,33,36. All four reports on second-line treatment were available as abstracts only 17,30,38,39.

Sixteen of the included trial reports disclosed funding, either wholly or in part, from pharmaceutical companies 7,8,10,11,15–17,20–22,27,30,31,39. Seven reported Debiopharm as the source of funding 7,8,10,11,20–22; one reported Pharmacia (Pfizer)/Sanofi–Synthelabo 15; two reported Aventis 16,27; one reported Aventis / Sanofi–Synthelabo 31; and three reported Sanofi–Synthelabo 17,30,39. Five of the reports disclosed a hospital, university, research group, or another non-industry entity as the source of funding 32–35,37, and twelve trials did not report the source of funding 9,12–14,18,19,23–26,36,38. The reported sources of funding for the two obtained meta-analyses were a research group grant 28 and partial industry funding 29.

### 4.2 Outcomes

#### 4.2.1 Phase iii RCTs of Oxaliplatin Combined with 5-FU and FA as First-line Therapy for Advanced Colorectal Cancer

Twenty-seven reports 7–16,18–27,31–37 of rcts on first-line treatment, representing seventeen individual trials 7–16, 31–37 were obtained (Table I). Six reports of these seventeen trials were available in abstract form only 9,12, 13,14,33,36. Results for the outcomes of interest follow.

##### 1-Year Survival

Only seven of the seventeen trials reported 1-year survival data 8,10,11,15,31,32,35. One of the trials 8 compared two regimens that both contained 5-fu, fa, and oxaliplatin: one in a standard infusion, and the other in a chronomodulated regimen. Both arms reported similar 1-year survival rates (66% vs. 63%, *p* > 0.05). Of the other six trials, only two detected statistically significant 1-year survival differences: one for folfox4 over ifl (71% vs. 58%, *p* = 0.002) 15, and another for oxafafu over irifafu (39% vs. 23%, *p* = 0.032) 31.

##### Response Rates

Only two of the seventeen trials did not report data on response rates 9,12. Nine of the 15 trials reported statistically significant differences (*p* < 0.05) in response rates between treatment arms: three for chronomodulated regimens of 5-fu, fa, and oxaliplatin over standard infusion regimens of the same agents 7,8,11; and the others for lv5fu2 and oxaliplatin over lv5fu2 alone 10; for fufox over fufa 13; for folfox4 over ifl and for irox over ifl 15; for both low- and high-dose oxafafu over irifafu 31; for folfoxiri over folfiri 33; and for oxafafu over fafu^34^. One trial that reported on response rates did not report a *p* value 36. Three trials reported no significant difference between treatment arms: folfox and folfiri 14, folfox4 and irox 15, and the crossover trial by Tournigand *et al.* 16 that compared the sequences folfiri→folfox6 and folfox6→folfiri.

##### Quality of Life

Only two of the seventeen rcts reported data on qol 10,34. Of these latter two trials, neither reported a significant difference in qol scores between the trial arms: lv5fu2 plus oxaliplatin versus lv5fu2 10, or oxafafu versus fafu 33.

As reported in the rcts located by the literature search, first-line treatment with oxaliplatin was associated with significantly more peripheral and sensory neuropathy and neutropenia beyond the adverse effects expected with the other drugs given in the regimen.

#### 4.2.2 Meta-analyses of First-line Trials

The meta-analysis reported by Grothey *et al.* 28 included the fully published or publicly presented results from seven rcts involving 3186 patients. It detected a significant 3.5-month increase in median survival (*p* = 0.0083) in patients who received a first-line combination therapy (either oxaliplatin/5-fu/fa or irinotecan/5-fu/fa) as compared with patients who received monotherapy. The results of the meta-analysis indicated that, for maximum overall survival benefit, patients on a first-line combination therapy containing oxaliplatin should be offered combination therapy with irinotecan as second-line treatment, and vice versa. It appears that second-line treatment may compensate for a less active first-line treatment, as evidenced by the fact that patients who had access to all three active drugs (oxaliplatin, irinotecan, and 5-fu) showed the longest overall survival. However, this conclusion is confounded by the fact that patients who lived longer were more likely to have been treated with all three drugs. In addition, patients with lower performance status may have been excluded from second-line treatments using oxaliplatin and irinotecan. Additionally, oxaliplatin was not available to all patients, especially in older trials. The meta-analysis detected longer median survival times in more recent trials—trials more likely to use oxaliplatin in first- and second-line treatment.

An abstract report by Lévi *et al.* 29 pooled the 7-year results of two previously discussed, but individually underpowered, trials 7,8. Both trials compared chronomodulated (cm) infusion to flat infusion in the first-line treatment of mcc. Significant benefits were detected in overall response rate (orr%: 51% vs. 30%; *p* < 0.001), complete surgical resection (23.3% vs. 12.8%, *p* < 0.001), and median progression-free survival (pfs: 10.3 months vs. 7.5 months; *p* = 0.039) favouring cm therapy; however, no difference was detected in median survival (18.6 months vs. 16.5 months, *p* = 0.22). Pooling the data from the two trials did not detect any difference in survival at either 5 or 7 years (5-year survival: 12.6% vs. 15.2%; 7-year survival: 6.6% vs. 7.1%). The results may have been confounded by an imbalance between the studies with regard to recurrent metastatic disease following surgery for liver metastases (10% of patients receiving flat infusion vs. 22% of patients receiving cm infusion, *p* < 0.001), and in addition, by significant treatment crossover from the flat infusion to the cm infusion arm (26% of patients), which affected median survival (14.7 months non-crossover vs. 18.5 months crossover; *p* = 0.043). The pooled results from those two trials confirmed that, as compared with flat infusion, cm infusion significantly improved the orr% and pfs.

#### 4.2.3 Phase III RCTs of Oxaliplatin Combined with 5-FU and FA as Second-line Therapy for Advanced Colorectal Cancer

Four reports 17,30,38,39 describing four rcts of second-line treatment were obtained (Table I). All of these trial reports were available in abstract form only.

##### 1-Year Survival

None of the second-line treatment reports obtained provided data on 1-year survival.

##### Response Rates

Three of the four reports 17,30,39 provided data on response rates. All three trials reported statistically significant differences between the trial arms, two for folfox4 over lv5fu2 17,30 (9.9% vs. 0%, *p* < 0.0001 30; 11.1% vs. 1.9%, *p* < 0.05 17), and the third for the sequence folfox4→cpt-11 over cpt-11→ folfox4 (27% vs. 15%, *p* < 0.0142)^39^.

##### Quality of Life

None of these abstract reports provided data on qol.

As indicated under the heading “Quality of Life” in subsection 4.2.1, treatment with oxaliplatin was associated with significantly more peripheral and sensory neuropathy and neutropenia beyond the adverse effects expected from other drugs given in the regimen.

## 5. INTERPRETIVE SUMMARY

The combination regimens using infusional 5-fu (folfox or folfiri) both represent acceptable treatment alternatives for first-line therapy in fit patients.

Oxaliplatin is active in colorectal cancer, and the evidence supports its use in combination with infusional 5-fu/fa (folfox). Oxaliplatin without 5-fu/fa does not appear to have meaningful activity. The folfox regimen has definite advantages over bolus ifl in terms of toxicity, objective response rate (45% vs. 31%), median ttp (8.7 months vs. 6.9 months), median survival (19.5 months vs. 15.0 months), and 1-year survival (71% vs. 58%) as demonstrated in the N9741 study 15.

The superior 1-year survival seen in the N9741 study may have two possible explanations:

- The 5-fu was given as an infusion in the folfox arm, but as a bolus in the ifl arm, and infusional 5-fu has demonstrated superiority over bolus 5-fu in terms of toxicity and tumour response rate. This fact alone may therefore account for the differences seen in the two regimens. It may also account for the lack of difference seen between folfox and folfiri in the gercor study, because both regimens used infusional 5-fu 16.
- Superior survival in the folfox arm may relate to the high rate of second-line irinotecan use. In the folfox arm, 53% of the patients received second-line irinotecan, but only 17% of patients on the ifl arm received second-line oxaliplatin. It is becoming increasingly clear that subsequent therapy can have a substantial effect on survival. From the evidence reviewed, it appears that the number of active drugs available to a study arm may positively affect survival, because when more drugs are made available to patients, median survival is increased. This finding does not rule out other factors, such as variations in study population and other variations in treatment over time, but it is highly supportive of the conclusion that access to all three active drugs (5-fu/fa, oxaliplatin, irinotecan) is important to optimize patient outcomes 27. It is also evident that when combination therapy is to be used, infusional rather than bolus 5-fu should be used. This recommendation to use 5-fu in an infusional schedule is now abundantly clear from single-agent studies, from combination studies in advanced disease, and from the adjuvant setting in early colorectal cancer. The role of bolus 5-fu in the management of colorectal cancer is becoming increasingly limited.

The combination of oxaliplatin and irinotecan is also active, but it has lower tumour response rates and 1-year survival rates than folfox does, and therefore no advantages 15.

The inconvenience of infusional 5-fu pump programs, in combination with the drug’s unavailability in certain regions, has led to interest in oral capecitabine as a possible replacement for infusional 5-fu in oxaliplatin and irinotecan combinations. The few phase ii trials reported thus far have demonstrated response rates and toxicity that appear comparable to those seen with infusional 5-fu combinations. In the future, capecitabine may supersede the 5-fu pump and the need for central venous access devices; however, this development will depend on the results of ongoing phase iii trials. Until the results of those trials are available, infusional 5-fu regimens—either alone or in combination—are standard therapy.

The question of whether to use a cm regimen of infusional 5-fu is a compelling area of study, but no such regimen has been widely evaluated outside of a few specialized centres. This question extends beyond a simple review of 5-fu/oxaliplatin combinations. The pooled analysis of the two underpowered studies by Lévi *et al.* 7,8 suggests that cm could both reduce toxicity and positively affect endpoints such as orr% and ttp. The intervention is worthy of further study, although the complexity of the therapy may put it beyond feasibility in many locales.

The ECF4584 trial 30 demonstrated improvements in response rate, ttp (median: 4.6 months vs. 1.6 months, *p* < 0.0001), and symptom control with second-line folfox as compared with oxaliplatin alone or infusional 5-fu/fa alone in patients who progressed on the ilf regimen. No overall survival analysis was performed. For patients who have progressed on both an anti-ts agent and irinotecan, folfox is the preferred therapy.

In patients with tumour progression on first-line 5-fu/fa, folfox is active, with an orr% that appears to compare favourably with that of folfiri, the standard regimen. Currently, more evidence supports second-line irinotecan than supports folfox or oxaliplatin alone, but for patients considered poor candidates for second-line irinotecan, folfox is a reasonable alternative. Further clarification of the role of oxaliplatin after progression on first-line 5-fu awaits more-mature data from the Sanofi-sponsored ECF4585 trial.

As treatment regimens for advanced colorectal cancer continue to evolve, recent trials 38,40 have investigated the addition of bevacizumab to the folfox regimen. An abstract report by Hochster *et al.* 40 of the tree-2 study—a first-line cohort study comparing folfox plus bevacizumab, oxafafu plus bevacizumab, and capecitabine and oxaliplatin (capox) plus bevacizumab—found that folfox plus bevacizumab resulted in the longest median survival with acceptable adverse events. In the tree-2 study, the median survivals for the treatment arms without bevacizumab were 19.2 months (folfox), 17.9 months (oxafafu), and 17.3 months (capox) as compared with 26 months (folfox + bevacizumab), 20.7 months (oxafafu + bevacizumab), and 27.0 months (capox + bevacizumab) 41. The overall median survival was 18.2 months (no bevacizumab) as compared with 24.4 months (bevacizumab added) 41. When the overall survival data from the tree-1 trial (no bevacizumab) were compared with the tree-2 trial data (bevacizumab added), the results were 12 months, 67.5% versus 79.1%; 18 months, 50.1% versus 64.7%; 24 months, 35.8% versus 50.7%—all in favour of the treatments including bevacizumab 41.

The second-line rct reported by Giantonio *et al.* 38, which compared folfox4 with and without bevacizumab, found that the addition of bevacizumab to the folfox regimen resulted in significant gains in both median survival (10.7 months vs. 10.2 months, *p* = 0.0024) and pfs (7.4 months vs. 5.5 months, *p* = 0.0003). Based on these two trials, we conclude that the addition of bevacizumab to an infusional 5-fu, fa, and oxaliplatin regimen may provide benefits beyond those that would be possible with infusional 5-fu, fa, and oxaliplatin without bevacizumab.

## 6. GASTROINTESTINAL CANCER DSG CONSENSUS PROCESS

### Note

The gi dsg consensus process was based on an earlier draft of the present document. That draft did not contain any of the evidence regarding the addition of bevacizumab to regimens of infusional 5-fu/fa plus oxaliplatin. References 15 and 31–41 were added to the document after the consensus process took place.

The systematic review found one first-line therapy trial 15 that demonstrated infusional 5-fu/fa/ oxaliplatin (folfox) to be superior to bolus 5-fu/fa/ irinotecan (ifl), with more favourable rates of median survival and tumour response. Compared with ifl, folfox has lower incidences of severe nausea, vomiting, diarrhea, and febrile neutropenia, but a higher incidence of peripheral neuropathy. Therefore, for first-line treatment, short-term infusional 5-fu/fa in combination with either oxaliplatin (folfox) or irinotecan (folfiri) is acceptable for fit patients when combination therapy is the preferred treatment. Choice of first-line therapy may rely on patient factors and preferences—for example, less neuropathy with irinotecan versus less alopecia with oxaliplatin.

For second-line treatment after progression on first-line anti-ts monotherapy (for example, 5-fu/fa, capecitabine), irinotecan is standard therapy. For patients with contraindications to the use of second-line irinotecan, folfox is a reasonable alternative. After progression on both irinotecan and an anti-ts agent, folfox is the preferred therapy.

The role of radiation therapy, either alone or in combination with chemotherapy, for locally advanced unresectable colorectal cancer was not addressed in this guideline. In addition, the use of cm regimens is a topic that intersects with the use of oxaliplatin/5-fu combinations, particularly cm 5-fu in those combinations. Chronomodulation of oxaliplatin has not been extensively studied and was not addressed, because the topic is beyond the scope of this guideline.

In conclusion, the gi dsg acknowledges that the combination of oxaliplatin with short-term infusional 5-fu and fa (folfox) is an important component of first- and second-line treatment of advanced colon cancer, and the dsg recommends that oxaliplatin be made available for the treatment of advanced colorectal cancer.

## 7. EXTERNAL REVIEW OF THE PRACTICE GUIDELINE REPORT

### Note

The practitioner feedback survey was based on an earlier draft of the present document. That draft did not contain any of the evidence regarding the addition of bevacizumab to infusional regimens of 5-fu/fa plus oxaliplatin. References 15 and 31–41 were added to the document after the practitioner feedback survey was complete.

Based on the evidence and the draft recommendations presented at the time of gi dsg consensus, feedback was sought from Ontario clinicians.

### 7.1 Methods

Practitioner feedback was obtained through a mailed survey of 63 practitioners in Ontario (11 medical oncologists, 9 radiation oncologists, 42 surgeons, and one other practitioner). The survey consisted of items evaluating the methods, results, and interpretive summary used to inform the draft recommendations and asking whether the draft recommendations should be approved as a practice guideline. Written comments were invited. The practitioner feedback survey was mailed September 15, 2004. Follow-up reminders were sent at 2 weeks (post card) and 4 weeks (complete package mailed again). The gi dsg reviewed the results of the survey.

### 7.2 Results

From among the 63 surveys distributed, 29 responses were received (46% response rate). “Responses” include returned completed surveys and telephone, fax, and e-mail responses. Of the practitioners who responded, 18 indicated that the report was relevant to their clinical practice, and they completed the survey. Table II summarizes key results of the practitioner feedback survey.

### 7.3 Summary of Written Comments

Four respondents (22%) provided written comments. The main points were these:

- Access issues with oxaliplatin are ongoing.
- The guidelines seem to be directed to the medical oncologists who provide the treatments. For other caregivers in the cancer system, this key message needs to be delivered: Cancer treatment is in a state of continuous development with increasing efficacy, therefore assessment by a medical oncologist is important for all patients.
- Some of the stated recommendations are currently in use and are being accepted with enthusiasm by both clinicians and patients.
- This guideline has not included any information regarding the role of radiation in the local management of rectal tumours. However, the guideline makes good sense with respect to the recommended chemotherapy regimens. Could a recommendation, or at least a comment, be added somewhere in the document regarding radiation timing, and radiation in combination with the recommended chemotherapy regimens?

### 7.4 Modifications/Actions

The gi dsg made the following modifications to the clinical practice guideline in response to the comments obtained during practitioner feedback:

- With respect to the ongoing access issues with oxaliplatin, the gi dsg acknowledges the major barrier that access represents to putting its recommendations into practice. The gi dsg hopes that their recommendation of oxaliplatin will raise awareness of the issue and facilitate the process of making this drug available to patients.
- With respect to the issue of radiation therapy, the gi dsg added a qualifying statement to both the abstract and the main document: “The role of radiation therapy, either alone or in combination with chemotherapy, for locally advanced unresectable colorectal cancer is not addressed in this guideline.”

## Figures and Tables

**TABLE I:** Phase III trials of oxaliplatin combined with 5-fluorouracil (5-fu) and folinic acid (fa) as first-line therapy for advanced colorectal cancer

*Reference*	*Regimen*	*Patients (*n*)*	*orr(%cr+pr)*	*Disease stabilization (%)*	*Medianttp(months)*	*Medianpfs(months)*	*Median survival (months)*	*Median follow-up time (months)*	*1-Year survival (%)*
First-line treatment									
Lévi *et al.*, 1994, France 7	5-fu/fa/oxaliplatin	47	32 (2+13)	45	nr	8	14.9	30	nr
	cm 5-fu/fa/oxaliplatin	45	53 (3+21)	33	nr	11	19		nr
			*p*=0.038	*p*=nr		*p*=nr	*p*=0.03		
Lévi *et al.*, 1997, France 8	5-fu/fa/oxaliplatin	93	29 (3+24)	nr	4.9	7.9	16.9	36	66
	cm 5-fu/fa/oxaliplatin	93	51 (5+42)	nr	6.4	9.8	15.9		63
			*p*<0.0001		*p*=0.006	*P*=NS	*p*=ns		*p*=ns
Buechele *et al.*, 2000,	5-fu/fa/oxaliplatin	nr	nr	nr	nr	nr	nr	nr	nr
Germany 9 (abstract)	5-fu/fa	nr	nr	nr	nr	nr	nr	nr	nr
de Gramont *et al.*, 2000,	lv5fu2/oxaliplatin	210	50.7 (3+102)	31.9	nr	9	16.2	27.7	69
France 10	lv5fu2	210	22.3 (1+45)	51.0	nr	6.2	14.7		61
			*p*=0.0001	*p*=nr		*p*=0.0001	*p*=0.05		*p*=ns
Giachetti *et al.*, 2000,	cm 5-fu/fa/oxaliplatin	100	53 (3+50)	24	nr	8.7	19.4	47	75^a^
France 11	cm 5-fu/fa	100	16 (0+16)	45	nr	6.1	19.9		71^a^
			*p*<0.0001	*p*=nr		*p*=0.48	*p*=ns		*p*=ns
Giachetti *et al.*, 2002,	cm 5-fu/fa/oxaliplatin	250	nr	nr	nr	nr	nr	nr	nr
France 12 (abstract, eortc trial)	5-fu/fa/oxaliplatin	250	nr	nr	nr	nr	nr	nr	nr
Grothey *et al.,* 2002 13 (abstract)	fufa (Mayo)	124	22.6	nr	nr	5.3	16.1	27.3	nr
	fufox	114	48.3	nr	nr	7.9	20.4		nr
			*p*<0.0001			*p*<0.0001	*p*=nr		
Colucci *et al.,* 2003 14 (abstract)	folfox	145	36	39	nr	nr	nr	nr	nr
	folfiri	178	34	44	nr	nr	nr	nr	nr
			*p*=ns	*p*=nr					
Goldberg *et al.,* 2004 15 (Intergroup N9741)	(A) folfox4	267	45	nr	8.7	nr	19.5	nr	71^a^
	(B) ifl	264	31	nr	6.9	nr	15.0	nr	58 a
	(C) irox	264	35	nr	6.5	nr	17.4	nr	65 a
			A vs. B: *p*<0.05		A vs. B: *p*<0.05		A vs. B: *p*<0.05		A vs. B: *p*=0.002
			B vs. C: *p*<0.05		B vs. C: *p*<0.05		B vs. C: *p*<0.05		B vs. C: *p*=ns
			A vs. C: *p*=ns		A vs. C: *p*=ns		A vs. C: *p*=ns		A vs. C: *p*=ns
Tournigand *et al.,* 2004 16 (gercor)	folfiri→folfox6	109	56 (3+58) 1st line	23 1st line	nr	8.5 1st line	21.5	43.9	nr
			15 (0+12) 2nd line	48 2nd line		4.2 2nd line			
	folfox6 →folfiri	111	54 (5+54) 1st line	27 1st line	nr	8.0 1st line	20.6		nr
			4 (0+3) 2nd line	30 2nd line		2.5 2nd line			
			*P*=NS	*p*=nr			*p*=ns		
Comella *et al.,* 2005 31 (sicog)	irifafu	135	31 (16+26)	27	7.9	5.8	15.6	24	23
	oxafafu high-dose	71	41 (7+22)	21	10.5		17.6		
	oxafafu low-dose	68	47 (13+19)	22	7.9	7.0	23+		39
			*p=*0.029						*p=*0.032
Colucci *et al.,* 2005 32 (goim)	folfiri	178	34 (8+48)	42	7	nr	14	31	55
	folfox4	182	36 (9+53)	38	7	nr	15		62
			*p=*0.60						*p>*0.05
Falcone *et al.,* 2006 33 (gono, abstract)	folfoxiri	122	66	nr	nr	9.8	22.6	15.2	nr
	folfiri	122	41	nr	nr	6.9	16.7		nr
			*p=*0.0002			*p=*0.0006	*p=*0.032		
Hospers *et al.,* 2006^34^	oxafafu	151	33.8	43	nr	6.7	13.8	31.8	nr
	fafu	151	18.5	50.3	nr	5.6	13.3		nr
			*p=*0.004						
Souglakos *et al.,* 2006^35^ (horg)	folfoxiri	137	43 (9+50)	nr	8.4	nr	21.5	26	67
	folfiri	146	33.6 (5+44)	nr	6.9	nr	19.5		64
			*p=*ns		*p=*ns		*p=*ns		*p=*ns
Stanculeanu *et al.,* 2006 36 (abstract)	folfox4	22	64 (2+12)	27.3	nr	nr	nr	nr	nr
	folfiri	18	44 (1+7)	44.4	nr	nr	nr	nr	nr
	irox	17	53 (2+7)	35.3	nr	nr	nr	nr	nr
			*p=*nr						
Tournigand *et al.,* 2006 37 (gercor)	folfox4	311	58.5	nr	nr	9	19.3	31	nr
	folfox7	309	59.2	nr	nr	8.7	21.2		nr
			*p=*ns						
Second-line treatment									
Rothenberg *et al.,* 2003 30 (abstract, EFC4584)	lv5fu2	256	0	nr	2.7	nr	nr	nr	nr
	Oxaliplatin	266	nr	nr	nr	nr	nr	nr	nr
	folfox4	267	9.9	nr	4.6	nr	nr	nr	nr
			*p*<0.0001		*p*<0.0001				
Garay *et al.,* 2003 17 (abstract)	folfox4	105	11.1	nr	4.9	nr	nr	nr	nr
	lv5fu2	101	1.9	nr	2.6	nr	nr	nr	nr
			*p*<0.05		*p*<0.05				
Giantonio *et al.,* 2005 38 (abstract, ecog)	(A) folfox4+bevacizumab	290	nr	nr	nr	7.4	12.5	nr	nr
	(B) folfox4	289	nr	nr	nr	5.5	10.7	nr	nr
	(C) Bevacizumab	243	nr	nr	nr	3.5	10.2	nr	nr
						A vs. B: *p=*0.0003	A vs. B: *p=*0.0024		
Pitot *et al.,* 2005 39 (abstract, N9841)	cpt-11→folfox4	245	15	nr	4.0	nr	14.7	nr	nr
	folfox4→cpt-11	246	27	nr	5.2	nr	13.5	nr	nr
			p<0.01						

a Estimated from survival curve.

orr = overall response rate; cr = complete response; pr = partial response; ttp = time to progression; pfs = progression-free survival; nr = not reported; cm = chronomodulated delivery rate; ns = nonsignificant; lv5fu2 = leucovorin calcium (fa), 5-fu; eortc = European Organization for Research and Treatment of Cancer; fufa = 5-fu, fa; fufox = 5-fu, fa, oxaliplatin; folfox (appended digit indicates regimen variation) = fa, 5-fu, oxaliplatin; folfiri = fa, 5-fu, irinotecan; ifl = irinotecan, 5-fu, leucovorin calcium (fa); irox = irinotecan, oxaliplatin; gercor = Groupe Coopérateur Multidisciplinaire en Oncologie; sicog = Southern Italy Cooperative Oncology Group; irifafu = irinotecan, fa, 5-fu; oxafafu = oxaliplatin, fa, 5-fu; goim = Gruppo Oncologico Italia Meridionale; folfoxiri = fa, 5-fu, oxaliplatin, irinotecan; gono = Gruppo Oncologico Nord Ovest; oxafafu = oxaliplatin, fa, 5-fu; fafu = fa, 5-fu; ecog = Eastern Cooperative Oncology Group; cpt-11 = irinotecan.

**TABLE II:** Practitioner responses to eight items on the practitioner feedback survey

	*Percentage who*		
*Item*	*Strongly agree or agree*	*Neither agree nor disagree*	*Strongly disagree or disagree*
The rationale for developing a clinical practice guideline, as stated in the “Choice of Topic” section of the report, is clear.	100	—	—
There is a need for a clinical practice guideline on this topic.	89	11	—
The literature search is relevant and complete.	78	22	—
The results of the trials described in the report are interpreted according to my understanding of the data.	89	11	—
The draft recommendations in this report are clear.	78	17	6
I agree with the draft recommendations as stated.	83	11	6
This report should be approved as a practice guideline.	89	6	6
	*Very likely or likely*	*Unsure*	*Not at all likely or unlikely*
If this report were to become a practice guideline, how likely would you be to make use of it in your own practice?	67	11	11

## Appendix

**APPENDIX A:** Treatment options (refer to Appendix B for recommended dosages and schedules)

First-line treatment alternatives	Second-line treatment alternatives
folfiri (combination 5-fu/fa/irinotecan)	folfox (combination 5-fu/fa/oxaliplatin), after first-line folfiri
folfox (combination 5-fu/fa/oxaliplatin)	folfiri (combination 5-fu/fa/irinotecan), after first-line folfox
de Gramont schedule (infusional 5-fu/fa)	folfox (combination 5-fu/fa/oxaliplatin), plus bevacizumab
Raltitrexed	Irinotecan alone
Capecitabine	

## Appendix

**APPENDIX B:** Dosing by trial

*Reference*	*Regimen (mg/m**2**daily, frequency)*
First-line treatment	
Lévi *et al.,* 1994 7 (iocc trial)	5-fu 600, fa 300, oxaliplatin 20, days 1–5, every 21 days (16-day intermission) via programmable pump Arm A: flat infusion Arm B: cm infusion (5-fu/fa peak at 04:00 hours; oxaliplatin peak at 16:00 hours)
Lévi *et al.,* 1997 8 (iocc trial)	5-fu 600, fa 300, oxaliplatin 20, days 1–5, every 21 days (16-day intermission) Arm A: flat infusion Arm B: cm infusion
Buechele *et al.,* 2000, Germany 9	Oxaliplatin 50, 2-hour infusion + fa 500, 2-hour infusion + 5-fu 2000, 24-hour infusion; days 1, 8, 15, and 22; every 36 days versus Bolus 5-fu/fa (Mayo Clinic regimen)
de Gramont *et al.,* 2000 10	Oxaliplatin 85, 2-hour infusion, day 1 + 5-fu 400 bolus, then 600 continuous infusion, days 1 and 2 + fa 200, continuous infusion, days 1 and 2, every 2 weeks versus 5-fu 400 bolus, then 600 continuous infusion, days 1 and 2 + fa 200, continuous infusion, days 1 and 2, every 2 weeks (lv5fu2 regimen)
Giacchetti *et al.,* 2000 11	Oxaliplatin 125, 6-hour infusion, day 1 + 5-fu 700 + fa 300, cm infusion; days 1–5; every 3 weeks versus 5-fu 700 + fa 300, cm infusion, days 1–5, every 3 weeks
Giacchetti *et al.,* 2002 12 (eortc trial)	Oxaliplatin 25, cm infusion (peak at 16:00), 5-fu 750, cm infusion (peak at 04:00), fa 300, cm infusion (peak at 04:00); all three drugs given daily for 4 days and repeated every 2 weeks versus Oxaliplatin 100, 2-hour infusion, day 1; 5-fu 1500, 22-hour infusion, daily for 2 days; fa 600, 2-hour infusion daily for 2 days; repeat every 2 weeks (folfox2)
Grothey *et al.,* 2002, Germany 13	Oxaliplatin 50, 2-hour infusion + 5-fu 2000, 24-hour infusion + fa 500, 24-hour infusion; days 1, 8, 15, and 22; every 5 weeks (fufox) versus 5-fu 425 bolus + fa 20, days 1–5, every 29 days (Mayo)
Colucci *et al.,* 2003 14 (goim trial)	Oxaliplatin 85, day 1; fa 100, 2-hour infusion, days 1 and 2; 5-fu 400 bolus, followed by 5-fu 600, 22-hour infusion, days 1 and 2; every 2 weeks versus Irinotecan 180, day 1; fa 100, 2-hour infusion, days 1 and 2; 5-fu 400 bolus, followed by 5-fu 600, 22-hour infusion, days 1 and 2; every 2 weeks
Goldberg *et al.,* 2004 15 (Intergroup N9741 trial)	Oxaliplatin 85, day 1, followed by 5 fu - 400 bolus + 600 22-hour infusion, days 1 and 2; fa 200, days 1 and 2; every 2 weeks (de Gramont folfox4) versus Irinotecan 125 + 5-fu 500 + fa 20; days 1, 8, 15, and 22; every 6 weeks (Saltz ifl) versus Oxaliplatin 85, day 1 + irinotecan 200, day 1; every 3 weeks (Wasserman irox)
Tournigand *et al.,* 2004^16^ (gercor trial)	First-line folfiri: irinotecan 180 2-hour infusion, day 1; fa 200 2-hour infusion, day 1; 5-fu 400 bolus, day 1; followed by 5-fu 2400–3000 48-hour infusion, day 2; every 2 weeks until progression, then follow with second-line folfox6 (as below) versus First-line folfox6: oxaliplatin 100 2-hour infusion, day 1; fa 200 2-hour infusion, day 1; 5-fu 400 bolus, day 1; followed by 5-fu 2400–3000 48-hour infusion, day 2; every 2 weeks until progression, then follow with second-line folfiri (as above)
Comella *et al.,* 2005 31 (sicog)	irifafu: irinotecan 200 intravenously, day 1; fa 250 intravenously, followed by 5-fu 850, day 2 versus High-dose oxafafu: oxaliplatin 100, day 1; followed by fa 250 and 5-fu 1050, day 2 versus Low-dose oxafafu: oxaliplatin 85, day 1; fa 250 and 5-fu 850, day 1
Colucci *et al.,* 2005 32 (goim)	folfiri: irinotecan 180, day 1; fa 100 2-hour infusion, 5-fu 400 bolus, followed by 5-fu 600 22-hour infusion, days 1 and 2 versus folfox4: oxaliplatin 85, day 1; irinotecan 180, day 1; fa 100 2-hour infusion, 5-fu 400 bolus, followed by 5-fu 600 22-hour infusion, days 1 and 2
Falcone *et al.,* 2006 33 (gono)	folfoxiri : oxaliplatin 85, day 1; irinotecan 165, day 1; 5-fu 3200 48-hour infusion starting on day 1; l-fa 200, day 1; every 2 weeks versus folfiri: irinotecan 180, day 1; l-lv 100, days 1 and 2; 5-fu 400 bolus, days 1 and 2; followed by 5-fu 600 22-hour infusion, days 1 and 2; every 2 weeks At progression on folfiri, a folfox regimen was recommended
Hospers *et al.,* 2006 34	oxafafu: oxaliplatin 85 2-hour infusion, fa 200 1-hour infusion, 5-fu 2600 24-hour infusion, day 1, every 2 weeks versusfafu: 5-fu 425, days 1–5; fa 20, days 1–5; every 4 weeks
Souglakos *et al.,* 2006 35 (horg)	folfoxiri: oxaliplatin 65, day 2; irinotecan 150, day 1; fa 200, days 2 and 3; 5- fu 400 bolus, followed by 5-fu 600 22-hour infusion, days 2 and 3 versus folfiri: irinotecan 180, day 1; fa 200, days 2 and 3; 5-fu 400 bolus, followed by 5-fu 600 22-hour infusion, days 2 and 3
Stanculeanu *et al.,* 2006 36	folfox 4: oxaliplatin 85, day 1; fa 200, days 1 and 2; 5- fu 400 bolus, followed by 5- fu 600 22-hour infusion, days 1 and 2; every 15 days versus folfiri: irinotecan 180, fa 400, 5-fu 400 bolus, followed by 5-fu 2400 46-hour infusion, every 15 days versus irox: irinotecan 300, day 1; oxaliplatin 85, day 2; every 3 weeks
Tournigand *et al.,* 2006 37 (gercor)	folfox 4: oxaliplatin 85 2-hour infusion, day 1; fa 2-hour infusion (either 100 l-lv or 200 dl-lv), 5-fu 400 bolus, followed by 5-fu 600 22-hour infusion, days 1 and 2; every 2 weeks versus folfox7 (6 cycles) → lv5fu2 (12 cycles) → folfox7 (6 cycles) folfox7: oxaliplatin 130 2-hour infusion, day 1; fa 2-hour infusion (either l-lv 200 or dl-lv 400), followed by 5-fu 2400 46-hour infusion; every 2 weeks lv5fu2: fa 2-hour infusion (either l-lv 200 or dl-lv 400), 5-fu 400 bolus, followed by 5-fu 3000 46-hour infusion, every 2 weeks
Second-line treatment	
Rothenberg *et al.,* 2003 30 (EFC4584 trial)	Treatment given as second-line to ifl Oxaliplatin 85 2-hour infusion, day 1; 5-fu 400 bolus, followed by 5-fu 600 22-hour infusion, days 1 and 2; every 2 weeks (folfox4) versus 5-fu 400 bolus, followed by 5-fu 600 22-hour infusion, days 1 and 2; fa 200; every 2 weeks (lv5fu2)
Garay *et al.,* 2003^17^ (Sanofi/Memorial Sloan Kettering Cancer Centre crossover trial)	Treatment given as second-line to 5-fu + irinotecan ± fa Oxaliplatin 85 2-hour infusion, day 1; 5-fu 400 bolus; followed by 5-fu 600 22-hour infusion, days 1 and 2; every 2 weeks (folfox4) versus 5-fu 400 bolus, followed by 5-fu 600 22-hour infusion, days 1 and 2; fa 200; every 2 weeks (lv5fu2)
Giantonio *et al.,* 2005 38 (ecog)	folfox 4 + bevacizumab: bevacizumab 10 mg/kg intravenously, biweekly; oxaliplatin 85, day 1; fa 200 2-hour infusion, 5-fu 400 bolus, followed by 5-fu 600 22-hour infusion, days 1 and 2 versus folfox4: oxaliplatin 85, day 1; fa 200 2-hour infusion, 5-fu 400 bolus, followed by 5-fu 600 22-hour infusion, days 1 and 2 versus Bevacizumab: bevacizumab 10 mg/kg intravenously, biweekly
Pitot *et al.,* 2005 39 (N9841)	Irinotecan → folfox4 versus folfox4 → irinotecan Irinotecan 350, day 1, every 3 weeks (reduced to 300 for ecog performance status 2, age ≥ 70, or prior pelvic radiation) folfox4: oxaliplatin 85, fa 200, 5-fu 400 bolus, followed by 600 22-hour infusion, days 1 and 2, every 2 weeks

iocc = International Organization Against Cancer; 5-fu = 5-fluorouracil; fa = folinic acid; cm = chronomodulated; eortc = European Organization for Research and Treatment of Cancer; goim = Gruppo Oncologico Italia Meridionale; gercor = Groupe Coopérateur Multidisciplinaire en Oncologie; sicog = Southern Italy Cooperative Oncology Group; l-fa = levo-folinic acid; gono = Gruppo Oncologico Nord Ovest; l-lv = levo-leucovorin calcium; horg = Hellenic Oncology Research Group; dl-lv = racemic leucovorin calcium; ecog = Eastern Cooperative Oncology Group.
